# Editorial: Xenotransplantation for the therapy of diabetes: A new look

**DOI:** 10.3389/fendo.2023.1190442

**Published:** 2023-03-31

**Authors:** Kazuhiko Yamada, Rita Bottino

**Affiliations:** ^1^ Johns Hopkins Medicine, Division of Transplant Surgery, Department of Surgery, Johns Hopkins University, Baltimore, MD, United States; ^2^ Imagine Islet Center - Imagine Pharma, Pittsburgh, PA, United States

**Keywords:** islet transplantation, xenotranplantation, islet replacement, blastocyst complementation, porcine islets

The Research Topic “*Xenotransplantation for the Therapy of Diabetes: A New Look*” represents a collection of mini-review articles and original research articles, which together describe the latest strategies and development directions of xeno islet transplantation and stem cells in type 1 diabetes (T1D) treatment.

The prevalence of type 1 diabetes mellitus (T1D) increased by 30% in the United States from 2017 to 2020 (1). Diabetes increases the incidence of ESRD, with the 30-year cumulative incidence ranging from 15% to 20% (2). The transplantation of allogeneic islets is a promising therapy for T1D. Despite the significant progress made over the past 20 years in free islet transplantation (Tx), this procedure typically requires pooling islets from multiple deceased pancreas donors in order to achieve glucose control (3, 4).

Even if recent advances, including more efficacious immunosuppressive protocols, have improved the outcome of islet Tx (5), the availability of allogeneic islets remains one of the major obstacles to the progress of allogeneic islet transplantation; this is made worse by the fact that as many as 50% of the cases reported by the recent phase three trial of islet transplantation require islets from multiple donors. Unfortunately, the number of usable pancreata from deceased human donors is far too small to provide sufficient islets to offer treatment to all patients who could benefit from allogeneic islet transplantation.

Amid progress in experimental studies over the past 5 years, xenotransplantation is becoming a more realistic strategy to address organ shortage (6). The editing of pig genes to fill the intra-species incompatibility gaps have further contributed to advanced interest in xenotransplantation. Using recent gene-editing technologies, xenotransplantation from multi-transgenic alpha-1,3-galactosyltransferase knockout pigs has demonstrated a marked prolongation of renal xenograft survival, ranging from days to greater than 6 months for islets, 1 year for life-supporting kidneys, and >2 years in a heterotopic non-life-supporting cardiac xenograft model (7–11). However, it is not clear which gene manipulations are essential for successful xenogeneic islet transplantation. In addition, although > 1-year survival of porcine islets in non-human primate models has been reported, continuous administration of multiple immunosuppressive drugs is required (12, 13), and recipients typically succumb to complications associated with chronic immunosuppression. Attempts to taper immunosuppression have been unsuccessful in islet xenoTx in preclinical models. Moreover, life-long multiple immunosuppressive drugs constitute a substantial limitation to the clinical application of islet xenoTx, providing a compelling rationale to pursue a clinically applicable strategy for the induction of tolerance.

The recent news from New York announcing kidney xenotransplantation in two brain death patients (14) and, subsequently, the world’s first Tx of a genetically modified pig heart in a human patient has caught the public’s attention (15). It is now timely to review and discuss the current status and potential of clinical islet xenoTx.

In the Research Topic entitled “*Xenotransplantation for the Therapy of Diabetes: A New Look*” we present four articles. Two of them focus on characterizing porcine islets and the other two introduce complementary or alternative strategies for islet replacement, such as *via* blastocyst complementation technology or human stem-cell-derived beta cells.

The article by Arefanian et al. from the University of Alberta assessed the yield, cell composition, and function of islets isolated from neonatal pigs at different ages. For previous experimental studies, two sources of pig islets have historically been used: neonatal pigs (16) or adult pigs (13, 17–19). It is generally accepted that adult pig islets are fragile and that their isolation is technically more challenging than human islet isolation; only a limited number of facilities are proficient in the provision of adult pig islets. Neonatal pig islet isolation is technically less complicated and therefore more broadly replicable. However, the islet yields are lower, thus, requiring multiple donors versus one adult to generate an islet mass sufficient to reverse diabetes in large mammals and potentially in human recipients. Neonatal islets compared to adult islets require a longer time to produce insulin following Tx (16). Thus, it is not yet clear whether to use neonate or adult pigs as islets donors and this decision, therefore, is subject to the individual institutions. Optimizing pig donor age for multi-organ retrieval for xenoTx poses a significant practical problem for companies seeking to raise pigs for xenotransplantation. Arefranian/Rayat et al. present a study that compares the functional performance of neonatal pig islets at ages, 3, 5, 7, or 10 days, with the aim to identify a preferable donor age. They concluded that islets from 7-day-old donors offer higher yields and better functions.

Most studies focusing on porcine islet physiology examine beta cell function and insulin secretion, but less is known about glucagon responses by porcine alpha cells. Mourad et al. at the University Catholique de Louvain assessed glucagon secretion by comparing neonatal porcine islets with adult porcine islets. Glucagon and insulin were assessed both *in vitro* through dynamic perifusion of isolated islets and *in vivo* with glucose tolerance tests. Porcine beta cells have been known to be less responsive to glucose stimulation than human beta cells; however, Mourad et al. demonstrated that porcine alpha cells are particularly responsive to glucose changes. These findings suggest the critical role of glucagon in porcine islet physiology and underscore the importance of characterizing species-specific differences in endocrine cell function. The combination of low insulin response to glucose by beta cells and the potent glucose-mediated inhibitory response of glucagon secretion by alpha cells may explain the supraphysiologic blood glucose levels in recipients after porcine islet transplantation even if adequate numbers of islets are transplanted.

The other studies included in this Research Topic introduce new and emerging strategies for islet replacement. Kano et al. describes progress led by his group and others in organogenesis using blastocyst complementation. Since it was first proposed in 2010, substantial technical improvements have led to notable successes in rat-to-mouse islet xenotransplantation (19). This technology is still in its infancy, and there are still challenges that limit the rapid translation of these results from small animal studies to large animal interspecies studies. While it is not clear that this approach will be applied to islet transplantation, given advances in genetic engineering of source animals, blastocyst complementation may be a particularly attractive strategy for the development and transplantation of complex organs such as the lungs and liver where current genetic modification strategies of the organs/tissues have proven insufficient.

Lastly, Naqvi et al. at the University of Illinois provides a comprehensive review of challenges and potential solutions in the field of islet xenotransplantation. In particular, this paper highlights an emerging (and directly applicable to xenotransplantation) strategy for the generation of human beta cells using iPSCs. If fully successful, this approach may theoretically obviate the need for xenotransplantation. The results of *in vitro* testing suggest that iPSC-derived beta cells function similarly to primary human islet cells; whether this function is preserved *in vivo* after transplantation is the subject of ongoing clinical trials. The prospect of using either human (allogeneic) or patient-derived (autologous) iPSCs is particularly attractive as overcoming xenogeneic barriers in pig-to-human islet transplantation remains a challenge. However, stem cell therapy is also a new field with its potential drawbacks including the theoretical risk of oncogenesis from the viral vector technology used to insert transcription factors. Both stem cell therapy and xenotransplantation remain, therefore, promising new technologies while requiring additional research.

## Author contributions

All the authors have made a substantial, direct, and intellectual contribution to the work and approved it for publication.
